# Supplementary material for: The Utility of Pre-Treatment Inflammation Markers as Associative Factors to the Adverse Outcomes of Vulvar Cancer: A Study on Staging, Nodal Involvement, and Metastasis Models
Source: J Clin Med. 2022 Dec 22;12(1):96. doi: 10.3390/jcm12010096 (PMC9821387; doi:10.3390/jcm12010096)
Supplement: Supplementary file 1 [file jcm-12-00096-s001.zip › 1. Table S1. More detailed analysis of the baseline laboratory data.pdf]

Table S1: More detailed analysis of the baseline laboratory data

Laboratory Profiles	Overall included cases	Mean ± standard deviation or Median (Interquartile range: 25–75% quartile)								
		Clinical staging			Lymph node metastasis (LNM)			Distant metastasis (DM)		
		Early stage/ I-II	Advanced stage/ III-IV	p-value	LNM (-)	LNM (+)	p-value	DM (-)	DM (+)	p-value
**Electrolytes**										
Sodium (mEq/L)*	136.5 (130.0-140.0)	138.0 (133.7-141.0)	136.0 (129.0-140.0)	0.088[a]	137.0 (128.5-140.0)	135.0 (130.0-140.0)	0.585[a]	137.0 (131.7-140.0)	134.5 (128.2-139.0)	0.141[a]
Potassium (mEq/L)*	3.9 (3.6-4.3)	3.9 (3.6-4.0)	3.9 (3.6-4.3)	0.389[a]	3.9 (3.6-4.2)	4.0 (3.5-4.4)	0.416[a]	3.9 (3.6-4.2)	3.9 (3.7-4.3)	0.355[a]
Chloride (mEq/L)*	101.0 (97.1-105.0)	104.0 (98.4-105.6)	100.2 (96.5-104.2)	0.119[a]	101.4 (96.9-105.7)	100.20 (97.15-103.80)	0.228[a]	102.2 (98.0-105.1)	99.0 (96.5-103.4)	0.114[a]
Calcium ions (mmol/L)*	1.2 (1.1-1.3)	1.2[c]	1.1 (1.1-1.3)	0.923[a]	1.2 (1.1-1.3)	1.15 (1.01-1.28)	0.556[a]	1.1 (1.0-1.2)	1.2 (1.1-1.3)	0.354[a]
Phosphate (mg/dL)*	3.3 ± 0.8	2.4[d]	3.7 (2.6-3.9)	0.113[a]	3.0 (2.4-3.9)	3.35 (2.45-3.87)	0.883[a]	3.2 ± 1.1	3.3 ± 0.6	0.744[b]
Magnesium (mg/dL)*	1.9 ± 0.3	1.6[d]	1.87 (1.7-2.3)	0.340[a]	1.8 (1.6-2.0)	1.93 (1.69-2.36)	0.277[a]	1.9 ± 0.3	2.0 ± 0.3	0.492[b]
**Chemistry – General**										
RBG (mg/dL)*	123.5 (100.7-171.2)	120.0 (101.5-160.7)	126.0 (100.0-181.2)	0.399[a]	118.0 (98.5-161.5)	135.00 (102.50-179.50)	0.160[a]	123.5 (100.7-168.0)	123.5 (98.2-175.0)	0.837[a]
Total bilirubin (mg/dL)**	0.4 (0.3-1.0)	0.4[c]	0.4 (0.3-1.6)	0.817[a]	0.4 (0.2-1.9)	0.44 (0.31-4.70)	0.624[a]	0.4 (0.2-0.6)	0.4 (0.3-3.5)	0.601[a]
Direct bilirubin (mg/dL)**	0.2 (0.1-0.4)	0.2[c]	0.2 (0.1-0.8)	0.728[a]	0.2 (0.1-1.1)	0.24 (0.10-3.34)	0.951[a]	0.1 (0.0-0.3)	0.2 (0.1-1.9)	0.102[a]
Indirect bilirubin (mg/dL)**	0.2 (0.1-0.6)	0.2[c]	0.2 (0.1-0.8)	0.643[a]	0.2 (0.1-0.9)	0.17 (0.12-1.36)	0.951[a]	0.2 (0.1-0.4)	0.2 (0.1-1.6)	0.489[a]
**Hemostasis and Clotting**										
PT ratio vs control (times)*	0.9 (0.9-1.0)	0.9 (0.9-1.0)	1.0 (0.9-1.0)	0.050[a]	0.9 (0.9-1.0)	0.96 (0.94-1.05)	0.087[a]	0.9 (0.9-1.0)	1.0 (0.9-1.0)	0.198[a]
APTT ratio vs control (times)*	1.0 (0.9-1.2)	1.0 (0.9-1.1)	1.1 (0.9-1.2)	0.111[a]	1.0 (0.9-1.1)	1.07 (0.93-1.16)	0.759[a]	1.0 (0.9-1.11)	1.1 (0.9-1.2)	0.424[a]
Fibrinogen (mg/dL)***	376.1 ± 168.2	164.5[c]	379.5 (247.4-520.6)	0.201[a]	358.7 (164.5-472.5)	418.30 (283.77-540.20)	0.398[a]	376.0 ± 156.0	376.1 ± 187.0	0.999[b]
D-Dimer (ng/mL)****	600.0 (200.0- 2,395.0)	800.0[c]	585.0 (150.0-2397.5)	0.741[a]	800.0 (300.0-3320.0)	585 (100-2172.50)	0.575[a]	1225.0 (540.0-2630.0)	360.0 (100.0-2100.0)	0.112[a]

[c]Mann-Whitney U test; [a]Student-t-test for equal variances assumed; [b]Student-t-test for equal variances not assumed; [c]only one case available; [d]only two cases available; *n=86; **n=15; ***n=19; ****n=21. **Abbreviations:** APTT, activated partial thrombin time; eGFR, estimated glomerular filtration rate; PT, prothrombin time; RBG, random blood glucose.
