# Supplementary material for: The Utility of Pre-Treatment Inflammation Markers as Associative Factors to the Adverse Outcomes of Vulvar Cancer: A Study on Staging, Nodal Involvement, and Metastasis Models
Source: J Clin Med. 2022 Dec 22;12(1):96. doi: 10.3390/jcm12010096 (PMC9821387; doi:10.3390/jcm12010096)
Supplement: Supplementary file 1 [file jcm-12-00096-s001.zip › 2. Table S2. Cutoff Data with Detailed Confidence Interval.pdf]

Table S2: Cut-offs and diagnostic indicators performance of inflammatory markers with detailed data of confidence intervals associated with clinical staging, lymph node metastasis, and distant metastasis

Clinical staging

Diagnostic performance indicators	Inflammatory surrogate markers																
	**LPR**	**NLR**	**dNLR**	**NMR**	**PLR**	**LMR**	**BLR**	**SII**	**BAN Score**	**HPR**	**ESR**	**PNI Score**	**mGPS**	**CRP**	**Procalcitonin**	**CRP/Alb Ratio**	**CRP/PCT Ratio**
Cut-off	22.70	2.83	2.075	15.765	202.14	2.205	0.035	1348.115	334.89	0.325	104	47.50	0.5	5.485	0.11	1.295	228.52
AUC (CI95%)	0.55 (0.40-0.70)	0.69 (0.54-0.84)	0.65 (0.50-0.81)	0.51 (0.07-0.92)	0.64 (0.49-0.78)	0.71 (0.57-0.85)	0.61 (0.47-0.75)	0.63 (0.48-0.78)	0.69 (0.53-0.84)	0.48 (0.33-0.63)	0.64 (0.49-0.78)	0.75 (0.62-0.88)	0.87 (0.74-0.99)	0.94 (0.86-1.00)	0.93 (0.81-1.00)	0.94 (0.86-1.00)	0.50 (0.19-0.81)
StdErr	0.075	0.075	0.078	0.075	0.074	0.072	0.070	0.078	0.079	0.077	0.076	0.066	0.066	0.042	0.063	0.042	0.158
p-value	0.510	0.014	0.047	0.920	0.075	**0.007**	0.158	0.087	**0.015**	0.795	0.076	**0.001**	0.018	**0.005**	**0.044**	**0.005**	1.000
Sensitivity	72.06% (59.85-82.27%)	86.76% (76.36-93.77%)	85.29% (74.61-92.72%)	23.53% (14.09-35.38%)	69.12% (56.74-79.76%)	67.65% (55.21-78.49%)	48.53% (36.22-60.97%)	76.47% (64.62-85.91%)	89.71% (79.93-95.76%)	52.94% (40.45-65.17%)	51.47% (39.03-63.78%)	80.88% (69.53-89.41%)	74.07% (53.72-88.89%)	92.59% (75.71-99.09%)	86.21% (68.34-96.11%)	92.6%	31.8% (13.86-54.87%)
Specificity	44.44% (21.53-69.24%)	55.56% (30.76-78.47%)	55.56% (30.76-78.47%)	88.89% (65.29-98.62%)	66.67% (40.99-86.66%)	77.78% (52.36-93.59%)	77.78% (52.36-93.59%)	55.56% (30.76-78.47%)	55.56% (30.76-78.47%)	55.56% (30.76-78.47%)	83.33% (58.58-94.42%)	61.11% (35.75-82.70%)	100% (39.76-100%)	100% (39.76-100%)	100% (15.81% (100%)	100%	100% (15.81-100%)
LR (+)	1.30 (0.84-2.01)	1.95 (1.16-3.30)	1.92 (1.13-3.25)	2.12 (0.54-8.38)	2.07 (1.06-4.06)	3.04 (1.26-7.34)	2.18 (0.89-5.36)	1.72 (1.01-2.93)	2.02 (1.20-3.40)	1.19 (0.68-2.09)	3.09 (1.07-8.90)	2.08 (1.15-3.75)	n/a	n/a	n/a	1.00	n/a
LR (-)	0.63 (0.33-1.19)	0.24 (0.11-0.50)	0.26 (0.13-0.54)	0.86 (0.70-1.06)	0.46 (0.29-0.75)	0.42 (0.27-0.64)	0.66 (0.47-0.93)	0.42 (0.23-0.77)	0.19 (0.08-0.42)	0.85 (0.52-1.37)	0.58 (0.42-0.80)	0.31 (0.17-0.58)	0.26 (0.14-0.49)	0.07 (0.02-0.28)	0.14 (0.06-0.34)	n/a	0.68 (0.51-0.91)
PPV*	83.05% (75.96-88.37%)	88.06% (81.36-92.57%)	87.88% (81.08-92.46%)	88.89 (66.91-96.94%)	88.68% (80.00-93.88%)	92.00% (82.67-96.25%)	89.19% (77.06-95.30%)	86.67% (79.23-91.72%)	88.41% (81.89-92.79%)	81.82% (71.93-88.77%)	92.11% (80.19-97.11%)	88.71% (81.32-93.41%)	100%	100%	100%	87.10%	100%
NPV*	29.63% (18.13-44.45%)	52.63% (34.75-69.87%)	50.00% (33.05-66.95%)	23.53% (19.96-27.51%)	36.36% (26.07-48.08%)	38.89% (29.42-49.28%)	28.57% (22.20-35.93%)	38.46% (25.63-53.13%)	58.82% (38.76-76.33%)	23.81% (16.15-33.65%)	31.25% (24.81-38.51%)	45.83% (31.45-60.95%)	36.36% (23.20%-51.95%)	66.67% (34.51-88.36%)	33.33% (16.76-55.40%)	n/a	11.76% (9.11-15.07%)
Accuracy*	66.28% (55.28-76.12%)	80.23% (70.25-88.04%)	79.07% (68.95-87.10%)	37.21% (27.02-48.30%)	68.60% (57.70-78.19%)	69.77% (58.92-79.21%)	54.65% (43.55-65.42%)	72.09% (61.38-81.23%)	82.56% (72.87-89.90%)	53.49% (42.41-64.32%)	58.14% (47.01-68.70%)	76.74% (66.39-85.18%)	77.42% (58.90-90.41%)	93.55% (78.58-99.21%)	87.10% (70.17-96.37%)	87.10%	37.50% (18.80-59.41%)
CUI (+)	0.598	0.764	0.750	0.209	0.613	0.622	0.433	0.663	0.793	0.433	0.474	0.718	0.741	0.926	0.862	0.871	0.318
CUI (-)	0.132	0.292	0.278	0.209	0.242	0.302	0.222	0.214	0.327	0.132	0.260	0.280	0.364	0.667	0.333	n/a	0.118

(*) These values are dependent on disease prevalence.

Lymph node metastasis

Diagnostic Indicators	Inflammatory surrogate markers																
	LPR	NLR	dNLR	NMR	PLR	LMR	BLR	SII	BAN Score	HPR	ESR	PNI Score	mGPS	CRP	Procalcitonin	CRP/Alb Ratio	CRP/PCT Ratio
Cut-off	24.65	2.83	2.075	14.315	248.985	1.89	0.045	1413.135	238.45	0.325	87.5	47.50	1.50	5.485	2.72	1.295	880.665
AUC (CI95%)	0.48 (0.36-0.61)	0.55 (0.43-0.67)	0.52 (0.40-0.65)	0.50 (0.37-0.62)	0.58 (0.46-0.70)	0.54 (0.42-0.67)	0.54 (0.42-0.66)	0.56 (0.44-0.69)	0.56 (0.43-0.68)	0.53 (0.41-0.66)	0.56 (0.44-0.68)	0.59 (0.47-0.71)	0.51 (0.30-0.72)	0.44 (0.22-0.67)	0.61 (0.41-0.81)	0.46 (0.23-0.68)	0.37 (0.14-0.60)
StdErr	0.063	0.063	0.064	0.063	0.062	0.063	0.063	0.063	0.063	0.064	0.063	0.062	0.106	0.116	0.103	0.116	0.118
p-value	0.789	0.421	0.749	0.986	0.202	0.497	0.531	0.310	0.371	0.613	0.344	0.165	0.921	0.606	0.293	0.691	0.284
Sensitivity	66.67% (51.05-80.00%)	88.89% (75.95-96.29%)	86.67% (73.21-94.95%)	33.33% (20.00-48.95%)	62.22% (46.54-76.23%)	60.00% (44.33-74.30%)	37.78% (23.77-53.46%)	75.56% (60.46-87.12%)	82.22% (67.95-92.00%)	60.00% (44.33-74.30%)	66.67% (51.05-80.00%)	82.22% (67.95-92.00%)	52.94% (27.81-77.02%)	94.12% (71.31-99.85%)	35.29% (14.21-61.67%)	94.12% (71.31-99.85%)	15.38% (1.92-45.45%)
Specificity	39.02% (24.20-55.50%)	34.10% (20.08-50.59%)	34.10% (20.08-50.59%)	78.05% (62.39-89.44%)	56.10% (39.75-71.53%)	60.98% (44.50-75.80%)	82.93% (67.94-92.85%)	41.46% (26.32-57.89%)	36.59% (22.12-53.06%)	58.54% (42.11-73.68%)	51.22% (35.13-67.12%)	39.02% (24.20-55.50%)	50.00% (23.04-76.96%)	35.71% (12.76-65.86%)	100% (76.84-100%)	35.71% (12.76-64.86%)	100% (71.51-100%)
LR (+)	1.09 (0.79-1.51)	1.35 (1.06-1.72)	1.32 (1.03-1.69)	1.52 (0.75-3.09)	1.42 (0.94-2.14)	1.54 (0.98-2.41)	2.21 (1.02-4.79)	1.29 (0.95-1.75)	1.30 (0.99-1.70)	1.45 (0.94-2.24)	1.37 (0.94-1.99)	1.35 (1.02-1.78)	1.06 (0.53-2.11)	1.46 (0.97-2.20)	n/a	1.46 (0.97-2.20)	n/a
LR (-)	0.85 (0.49-1.50)	0.33 (0.13-0.82)	0.39 (0.17-0.92)	0.85 (0.66-1.11)	0.67 (0.42-1.07)	0.66 (0.43-1.01)	0.75 (0.57-0.98)	0.59 (0.31-1.11)	0.49 (0.23-1.03)	0.68 (0.44-1.06)	0.65 (0.39-1.08)	0.46 (0.22-0.95)	0.94 (0.45-1.95)	0.16 (0.02-1.25)	0.65 (0.46-0.92)	0.16 (0.02-1.25)	0.85 (0.67-1.07)
PPV	54.55% (46.55-62.31%)	59.70% (53.73-65.40%)	59.09% (52.98-64.93%)	62.50% (45.04-77.22%)	60.87% (50.69-70.18%)	62.79% (51.81-72.59%)	70.83% (52.88-84.01%)	58.62% (51.04-65.81%)	58.73% (52.09-65.07%)	61.36% (50.69-71.05%)	60.00% (50.75-68.59%)	59.68% (52.80-66.20%)	56.25% (39.22-71.92%)	64.00% (54.17-72.78%)	100%	64.00% (54.17-72.78%)	100%
NPV	51.61% (37.79-65.20%)	73.68% (52.51-87.64%)	70.00% (49.74-84.62%)	51.61% (45.06-58.11%)	57.50% (46.00-68.24%)	58.14% (47.37-68.18%)	54.84% (48.19-61.32%)	60.71% (45.16-74.36%)	65.22% (47.06-79.82%)	57.14% (46.18-67.45%)	58.33% (45.68-69.98%)	66.67% (48.94-80.67%)	46.67% (29.72-64.42%)	83.33% (39.71-97.43%)	56.00% (47.25-64.39%)	83.33% (39.71-97.43%)	50.00% (44.23-55.77%)
Accuracy	53.49% (42.41-64.32%)	62.79% (51.70-72.98%)	61.63% (50.51-71.92%)	54.65% (43.55-65.42%)	59.30% (48.17-69.78%)	60.47% (49.34-70.85%)	59.30% (48.17-69.78%)	59.30% (48.17-69.78%)	60.47% (49.34-70.85%)	59.30% (48.17-69.78%)	59.30% (48.17-69.78%)	61.63% (50.51-71.92%)	51.61% (33.06-69.85%)	67.74% (48.63-83.32%)	64.52% (45.37-80.77%)	67.74% (48.63-83.32%)	54.17% (32.82-74.45%)
CUI (+)	0.364	0.531	0.512	0.208	0.379	0.377	0.268	0.443	0.483	0.368	0.400	0.491	0.298	0.602	0.353	0.602	0.154
CUI (-)	0.201	0.252	0.239	0.403	0.323	0.355	0.455	0.252	0.239	0.334	0.299	0.260	0.233	0.298	0.560	0.298	0.500

(*) These values are dependent on disease prevalence.

Distant metastasis

Diagnostic performance indicators	Inflammatory surrogate markers																
	**LPR**	**NLR**	**dNLR**	**NMR**	**PLR**	**LMR**	**BLR**	**SII**	**BAN Score**	**HPR**	**ESR**	**PNI Score**	**mGPS**	**CRP**	**Procalcitonin**	**CRP/Alb Ratio**	**CRP/PCT Ratio**
Cut-off	34.15	5.67	3.455	9.535	223.965	2.34	0.035	1348.115	183.84	0.235	84	43.50	0.50	164.4	0.16	53.245	122.525
AUC (CI95%)	0.52 (0.38-0.65)	0.59 (0.46-0.71)	0.56 (0.43-0.69)	0.45 (0.33-0.58)	0.54 (0.41-0.67)	0.63 (0.51-0.75)	0.65 (0.53-0.77)	0.55 (0.43-0.68)	0.60 (0.47-0.72)	0.53 (0.40-0.66)	0.63 (0.51-0.75)	0.60 (0.48-0.72)	0.55 (0.35-0.76)	0.68 (0.49-0.87)	0.56 (0.34-0.78)	0.68 (0.49-0.87)	0.51 (0.26-0.75)
StdErr	0.067	0.064	0.065	0.064	0.065	0.061	0.063	0.064	0.062	0.067	0.060	0.062	0.105	0.098	0.112	0.097	0.125
p-value	0.806	0.169	0.360	0.464	0.514	**0.039**	**0.020**	0.432	0.133	0.636	**0.047**	0.131	0.621	0.082	0.540	0.086	0.954
Sensitivity	56.25% (37.66-73.64%)	68.75% (49.99-83.88%)	65.62% (46.81-81.43%)	65.62% (46.81-81.43%)	68.75% (49.99-83.88%)	81.25% (63.56-92.79%)	65.62% (46.81-81.43%)	84.38% (67.21-94.72%)	84.38% (67.21-94.72%)	37.50% (21.10-56.31%)	84.38% (67.21-94.72%)	75.00% (56.60-88.54%)	73.33% (44.90-92.21%)	46.67% (21.27-73.41%)	93.33% (68.05-99.83%)	46.67% (21.27-73.41%)	66.67% (34.89-90.08%)
Specificity	62.96% (48.74-75.71%)	53.70% (39.61-67.38%)	53.70% (39.61-67.38%)	38.89% (25.92-53.12%)	48.15% (34.34-62.16%)	48.15% (34.34-62.16%)	70.37% (56.39-82.02%)	38.89% (25.92-53.12%)	40.74% (27.57-54.97%)	77.78% (64.40-87.96%)	46.30% (32.62-60.39%)	48.15% (34.34-62.16%)	43.75% (19.75-70.12%)	93.75% (69.77-99.84%)	43.75% (19.75-70.12%)	87.50% (61.65%-98.45%)	58.33% (27.67-84.83%)
LR (+)	1.52 (0.96-2.41)	1.49 (1.03-2.15)	1.42 (0.97-2.08)	1.07 (0.77-1.49)	1.33 (0.94-1.88)	1.57 (1.15-2.13)	2.21 (1.37-3.58)	1.38 (1.06-1.79)	1.42 (1.09-1.86)	1.69 (0.86-3.30)	1.57 (1.18-2.10)	1.45 (1.04-2.00)	1.30 (0.77-2.21)	7.47 (1.04-53.72)	1.66 (1.06-2.61)	3.73 (0.92-15.21)	1.60 (0.73-3.49)
LR (-)	0.69 (0.45-1.08)	0.58 (0.33-1.03)	0.64 (0.37-1.10)	0.88 (0.49-1.58)	0.65 (0.36-1.16)	0.39 (0.18-0.84)	0.49 (0.29-0.81)	0.40 (0.17-0.96)	0.38 (0.16-0.91)	0.80 (0.59-1.09)	0.34 (0.14-0.79)	0.52 (0.27-1.01)	0.61 (0.22-1.67)	0.57 (0.35-0.93)	0.15 (0.02-1.10)	0.61 (0.37-1.01)	0.57 (0.22-1.45)
PPV	47.37% (36.16-58.85%)	46.81% (37.80-56.03%)	45.65% (36.45-55.16%)	38.89% (31.41-46.93%)	44.00% (35.70-52.65%)	48.15% (40.61-55.78%)	56.76% (44.78-67.99%)	45.00% (38.69-51.48%)	45.76% (39.25-52.42%)	50.00% (33.85-66.15%)	48.21% (41.08-55.42%)	46.15% (38.23-54.28%)	55.00% (41.86-67.47%)	87.50% (49.31-98.05%)	60.87% (49.73-70.98%)	77.78% (46.21-93.45%)	61.54% (42.31-77.73%)
NPV	70.83% (60.93-79.09%)	74.36% (62.11-83.69%)	72.50% (60.60-81.88%)	65.63% (51.57%-77.39%)	72.22% (59.19-82.34%)	81.25% (66.68-90.37%)	77.55% (67.49-85.18%)	80.77% (63.72-90.94%)	81.48% (64.90-91.28%)	67.74% (60.78-74.00%)	83.33% (68.02-92.16%)	76.47% (62.66-86.29%)	63.64% (39.01-82.72%)	65.22% (53.46-75.37%)	87.50% (49.31-98.05%)	63.64% (51.28-74.42%)	63.64% (40.79-81.63%)
Accuracy	60.47% (49.34-70.85%)	59.30% (48.17-69.78%)	58.14% (47.01-68.70%)	48.84% (37.90-59.86%)	55.81% (44.70-66.52%	60.47% (49.34-70.85%)	68.60% (57.70-78.19%)	55.81% (44.70-66.52%)	56.98% (45.85-67.61%)	62.79% (51.70-72.98%)	60.47% (49.34-70.85%)	58.14% (47.01-68.70%	58.06% (39.08-75.45%)	70.97% (51.96-85.78%)	67.74% (48.63-83.32%)	67.74% (48.63-83.32%)	62.50% (40.59-81.20%)
CUI (+)	0.266	0.322	0.300	0.255	0.303	0.391	0.372	0.380	0.386	0.188	0.407	0.346	0.403	0.408	0.568	0.363	0.410
CUI (-)	0.446	0.399	0.389	0.255	0.348	0.391	0.546	0.314	0.332	0.527	0.386	0.368	0.278	0.611	0.383	0.557	0.371

(*) These values are dependent on disease prevalence.

Notes:

- The ‘sensitivity’ is the probability that a test result will be positive when the disease is present (true positive rate).
- Specificity is the probability that a test result will be negative when the disease is not present (true negative rate).
- The positive likelihood ratio is the ratio between the probability of a positive test result given the presence of the disease and the probability of a positive test result given the absence of the disease [i.e., True positive rate / False positive rate = Sensitivity / (1-Specificity)].
- The negative likelihood ratio is a ratio between the probability of a negative test result given the presence of the disease and the probability of a negative test result given the absence of the disease [i.e. = False negative rate / True negative rate = (1-Sensitivity) / specificity].
- Positive predictive value is probability that the disease is present when the test is positive [i.e., = sensitivity x prevalence / (sensitivity x prevalence) + (1-specificity) x (1-prevalence)].
- Negative predictive value is probability that the disease is not present when the test is negative [i.e., = specificity x (1-prevalence) / (1-sensitivity) x prevalence + specificity x (1-prevalence)].
- Last, accuracy indicates an overall probability that a patient is correctly classified, with the formula: Sensitivity × Prevalence + Specificity × (1 − Prevalence) [1].

Categorisation of AUC [2]**.**

Value	Interpretation
0.9–1	Excellent
0.8–0.9	Very good
0.7–0.8	Good
0.6–0.7	Sufficient
0.5–0.6	Poor
<0.5	Test not useful

Categorisation of sensitivity, specificity, PPV, NPV, and accuracy [3]

Value	Interpretation
≥95%	Excellent
80%–95%	Good
70%–80%	Moderate
<70%	Poor

Categorisation of clinical utility index [4,5]**.**

Value	Interpretation
≥0.81	Excellent utility
0.64-0.81	Good utility
0.49-0.64	Fair utility
0.36-0.49	Poor utility
<0.36	Very poor utility

Categorisation of likelihood ratio [6]

The more the likelihood ratio for a positive test (LR+) is greater than 1, the more likely the disease or outcome. The more a likelihood ratio for a negative test is less than 1, the less likely the disease or outcome

Value	Interpretation
>10	Large and often conclusive increase in the likelihood of disease
5 – 10	Moderate increase in the likelihood of disease
2 – 5	Small increase in the likelihood of disease
1 – 2	Minimal increase in the likelihood of disease
1	No change in the likelihood of disease
0.5 – 1.0	Minimal decrease in the likelihood of disease
0.2 – 0.5	Small decrease in the likelihood of disease
0.1 – 0.2	Moderate decrease in the likelihood of disease
< 0.1	Large and often conclusive decrease in the likelihood of disease

References:

1. MedCalc Software Ltd Diagnostic Test Evaluation Calculator v.20.114 Available online: https://www.medcalc.org/calc/diagnostic_test.php (accessed on 1 August 2022).
2. Šimundić, A.-M. Measures of Diagnostic Accuracy: Basic Definitions. *EJIFCC* **2009**, *19*, 203–211.
3. De, J.; Wand, A.P.F. Delirium Screening: A Systematic Review of Delirium Screening Tools in Hospitalized Patients. *Gerontologist* **2015**, *55*, 1079–1099, doi:10.1093/geront/gnv100.
4. Mitchell, A.J.; Pezzullo, J. Clinical Utility Index Calculator (CUI+ CUI-) v5: Quickly Find the Qualitative and Quantitative Accuracy & Utility of Diagnostic Predictive and Screening Tests Available online: https://www.psycho-oncology.info/cui.html (accessed on 1 August 2022).
5. Mitchell, A.J. Sensitivity × PPV Is a Recognized Test Called the Clinical Utility Index (CUI+). *European journal of epidemiology* 2011, *26*, 251–252; author reply 252.
6. American Academy of Family Physicians Likelihood Ratios, Predictive Values, and Post-Test Probabilities Available online: https://www.aafp.org/dam/AAFP/documents/journals/afp/Likelihood_Ratios.pdf (accessed on 21 August 2022).
