# Supplementary material for: The Utility of Pre-Treatment Inflammation Markers as Associative Factors to the Adverse Outcomes of Vulvar Cancer: A Study on Staging, Nodal Involvement, and Metastasis Models
Source: J Clin Med. 2022 Dec 22;12(1):96. doi: 10.3390/jcm12010096 (PMC9821387; doi:10.3390/jcm12010096)
Supplement: Supplementary file 1 [file jcm-12-00096-s001.zip › 3. Table S3. Details bivariate and multivariate analysis of staging models.pdf]

Table S3: Detailed performance of inflammatory markers using their tailored cut-offs associated with clinical staging in bivariate and multivariate analysis

Inflammatory markers	Clinical staging		Total	Bivariate analysis		Multivariate analysis	
	Advance	Early		Unadjusted OR (95%CI)	p-value	Adjusted OR (95%CI)	p-value
**LPR**							
High (≥22.70)	49 (72.1%)	10 (55.6%)	59 (68.6%)	2.06 (0.71-6.01)	0.180[b,c]	1.04 (0.20-5.40)	0.961[d]
Low (<22.70)	19 (27.9%)	8 (44.4%)	27 (31.4%)	Ref		Ref	
**NLR**							
High (≥2.83)	59 (86.8%)	8 (44.4%)	67 (77.9%)	8.19 (2.56-26.26)	**<0.0001[a,c]**	2.71 (0.19-38.14)	0.460[d]
Low (<2.83)	9 (13.2%)	10 (55.6%)	19 (22.1%)	Ref		Ref	
**dNLR**							
High (≥2.075)	58 (85.3%)	8 (44.4%)	66 (76.7%)	7.25 (2.30-22.82)	**0.001[a,c]**	Not Defined (0)	>0.999[d]
Low (<2.075)	10 (14.7%)	10 (55.6%)	20 (23.3%)	Ref			
**NMR**							
High (≥15.765)	16 (23.5%)	2 (11.1%)	18 (20.9%)	2.46 (0.51-11.87)	0.339[a]	**Not analysed**	
Low (<15.765)	52 (76.5%)	16 (88.9%)	68 (79.1%)	Ref			
**PLR**							
High (≥202.14)	47 (69.1%)	6 (33.3%)	53 (61.6%)	4.48 (1.48-13.54)	**0.006[b,c]**	1.93 (0.43-8.65)	0.389[d]
Low (<202.14)	21 (30.9%)	12 (66.7%)	33 (38.4%)	Ref		Ref	
**LMR**							
Low (≤2.205)	46 (67.6%)	4 (22.2%)	50 (58.1%)	7.32 (2.16-24.83)	**0.001[b,c]**	3.80 (0.77-18.70)	0.100[d]
High (>2.205)	22 (32.4%)	14 (77.8%)	36 (41.9%)	Ref		Ref	
**BLR**							
High (≥0.035)	33 (48.5%)	4 (22.2%)	37 (43.0%)	3.33 (1.00-11.05)	**0.045[b,c]**	0.73 (0.12-4.64)	0.741[d]
Low (<0.035)	35 (51.5%)	14 (77.8%)	49 (57.0%)	Ref		Ref	
**SII**							
High (≥1348.115)	52 (76.5%)	8 (44.4%)	60 (69.8%)	4.06 (1.37-12.03)	**0.009[b,c]**	Not Defined (0)	>0.999[d]
Low (<1348.115)	16 (23.5%)	10 (55.6%)	26 (30.2%)	Ref			
**BAN Score**							
Low (≤334.89)	61 (89.7%)	8 (44.4%)	69 (80.2%)	10.89 (3.23-36.71)	**<0.0001[a,c]**	9.20 (2.61-32.45)	**0.001[d]**
High (>334.89)	7 (10.3%)	10 (55.6%)	17 (19.8%)	Ref		Ref	
**HPR**							
Low (≤0.325)	36 (52.9%)	8 (44.4%)	44 (51.2%)	1.41 (0.49-4.00)	0.521[b]	**Not analysed**	
High (>0.325)	32 (47.1%)	10 (55.6%)	42 (48.8%)	Ref			
**ESR**							
High (≥104)	35 (51.5%)	3 (16.7%)	38 (44.2%)	5.30 (1.41-20.00)	**0.008[b,c]**	4.18 (1.01-17.32)	**0.048[d]**
Low (<104)	33 (48.5%)	15 (83.3%)	48 (55.8%)	Ref		Ref	
**PNI Score**							
Low (≤47.50)	55 (80.9%)	7 (38.9%)	62 (72.1%)	6.65 (2.16-20.46)	**<0.0001[b,c]**	1.43 (0.10-20.90)	0.794[d]
High (>47.50)	13 (19.1%)	11 (61.1%)	24 (27.9%)	Ref		Ref	
**mGPS**							
High (1-2)	20 (74.1%)	0	20 (64.5%)	n/a	**0.010[a]**	**Not analysed**	
Low (0)	7 (25.9%)	4 (100%)	11 (35.5%)				
**CRP**							
High (≥5.485)	25 (92.6%)	0	25 (80.6%)	n/a	**<0.0001[a]**	**Not analysed**	
Low (<5.485)	2 (7.4%)	4 (100%)	6 (19.4%)				
**Procalcitonin**							
High (≥0.11)	25 (86.2%)	0	25 (80.6%)	n/a	**0.032[a]**	**Not analysed**	
Low (<0.11)	4 (13.8%)	2 (100%)	6 (19.4%)				
**CRP/Alb Ratio**							
High (≥1.295)	27 (100%)	4 (100%)	31 (100%)	n/a	n/a	**Not analysed**	
Low (<1.295)	0	0	0				
**CRP/PCT Ratio**							

High (≥228.52)	7 (31.8%)	0	7 (29.2%)	n/a	>0.999[a]	**Not analysed**	
Low (<228.52)	15 (68.2%)	2 (100%)	17 (70.8%)				

[a]Fisher's exact test; [b]$\chi 2$ test; OR was obtained from the Mantel-Haenszel common odds ratio estimate; [c]variables with p-value ≤0.25 was eligible to enter multivariate analysis after bivariate analysis, except variables with n/a results for their OR; [d]multivariate analysis using the backward model; "n/a (not applicable)" denoted incalculably OR due to the presence of invalid (null) data in the 2 x 2 table; percent values (%) were calculated as a percentage of the column total.

Abbreviations: BAN, body mass index, albumin and neutrophil-lymphocyte ratio; BLR, basophil-to-monocyte ratio; CRP, C-reactive protein; CRP/Alb ratio, C-reactive protein-to-albumin ratio; CRP/PCT ratio, C-reactive protein-to-procalcitonin ratio; dNLR, derived neutrophil-to-lymphocyte ratio; ESR, erythrocyte sedimentation rate; HPR, haemoglobin-to-platelet ratio; LMR, lymphocyte-to-monocyte ratio; LPR, leukocyte-to-platelet ratio; mGPS, modified Glasgow Prognostic Score; NLR, neutrophil-to-lymphocyte ratio; NMR, neutrophil-to-monocyte ratio; PCT, procalcitonin; PLR, platelet-to-lymphocyte ratio; PNI, prognostic nutritional index; Ref, reference; SII, systemic immune-inflammation index.
