# Supplementary material for: The Utility of Pre-Treatment Inflammation Markers as Associative Factors to the Adverse Outcomes of Vulvar Cancer: A Study on Staging, Nodal Involvement, and Metastasis Models
Source: J Clin Med. 2022 Dec 22;12(1):96. doi: 10.3390/jcm12010096 (PMC9821387; doi:10.3390/jcm12010096)
Supplement: Supplementary file 1 [file jcm-12-00096-s001.zip › 4. Table S4. Details bivariate and multivariate analysis of LNM models.pdf]

Table S4: Detailed performance of inflammatory markers using their tailored cut-offs associated with lymph node metastasis in bivariate and multivariate analysis

Inflammatory markers	Lymph node metastasis		Total	Bivariate analysis		Multivariate analysis	
	LNM (+)	LNM (-)		Unadjusted OR (95%CI)	p-value	Adjusted OR (95%CI)	p-value
**LPR**							
High (≥24.65)	30 (66.7%)	25 (61.0%)	55 (64.0%)	1.28 (0.53-3.09)	0.583[a]	**Not analysed**	
Low (<24.65)	15 (33.3%)	16 (39.0%)	31 (36.0%)	Ref			
**NLR**							
High (≥2.83)	40 (88.9%)	27 (65.9%)	67 (77.9%)	4.15 (1.34-12.87)	**0.010**[a,c]	4.15 (1.34-12.86)	**0.014**[d]
Low (<2.83)	5 (11.1%)	14 (34.1%)	19 (22.1%)	Ref			
**dNLR**							
High (≥2.075)	39 (86.7%)	27 (65.9%)	66 (76.7%)	3.37 (1.15-9.87)	**0.022**[a,c]	Not Defined (0)	>0.999[d]
Low (<2.075)	6 (13.3%)	14 (34.1%)	20 (23.3%)	Ref			
**NMR**							
High (≥14.315)	15 (33.3%)	9 (22.0%)	24 (27.9%)	1.78 (0.68-4.67)	0.240[a,c]	1.25 (0.38-4.19)	0.712[d]
Low (<14.315)	30 (66.7%)	32 (78.0%)	62 (72.1%)	Ref			
**PLR**							
High (≥248.985)	28 (62.2%)	18 (43.9%)	46 (53.5%)	2.10 (0.89-4.98)	0.089[a,c]	1.00 (0.29-3.50)	0.989[d]
Low (<248.985)	17 (37.8%)	23 (56.1%)	40 (46.5%)	Ref			
**LMR**							
Low (≤1.89)	27 (60.0%)	16 (39.0%)	43 (50.0%)	2.34 (0.99-5.57)	0.052[a,c]	1.74 (0.50-6.08)	0.387[d]
High (>1.89)	18 (40.0%)	25 (61.0%)	43 (50.0%)	Ref			
**BLR**							
High (≥0.045)	17 (37.8%)	7 (17.1%)	24 (27.9%)	2.95 (1.07-8.11)	**0.033**[a,c]	2.05 (0.70-6.03)	0.192[d]
Low (<0.045)	28 (62.2%)	34 (82.9%)	62 (72.1%)	Ref			
**SII**							
High (≥1413.135)	34 (75.6%)	24 (58.5%)	58 (67.4%)	2.19 (0.87-5.50)	0.093[a,c]	0.39 (0.09-1.77)	0.222[d]
Low (<1413.135)	11 (24.4%)	17 (41.5%)	28 (32.6%)	Ref			
**BAN Score**							
Low (≤238.45)	37 (82.2%)	26 (63.4%)	63 (73.3%)	2.67 (1.00-7.21)	**0.049**[a,c]	0.31 (0.02-3.82)	0.359[d]
High (>238.45)	8 (17.8%)	15 (36.6%)	23 (26.7%)	Ref			
**HPR**							
Low (≤0.325)	27 (60.0%)	17 (41.5%)	44 (51.2%)	2.12 (0.89-5.01)	0.086[a,c]	2.00 (0.82-4.90)	0.127[d]
High (>0.325)	18 (40.0%)	24 (58.5%)	42 (48.8%)	Ref			
**ESR**							
High (≥87.5)	30 (66.7%)	20 (48.8%)	50 (58.1%)	2.10 (0.88-5.02)	0.093[a,c]	1.77 (0.69-4.55)	0.237[d]
Low (<87.5)	15 (33.3%)	21 (51.2%)	36 (41.9%)	Ref			
**PNI Score**							
Low (≤47.50)	37 (82.2%)	25 (61.0%)	62 (72.1%)	2.96 (1.10-7.96)	**0.028**[a,c]	1.26 (0.25-6.29)	0.780[d]
High (>47.50)	8 (17.8%)	16 (39.0%)	24 (27.9%)	Ref			
**mGPS**							
High (2)	9 (52.9%)	7 (50.0%)	16 (51.6%)	1.12 (0.27-4.63)	0.870[a]	**Not analysed**	
Low (0-1)	8 (47.1%)	7 (50.0%)	15 (48.4%)	Ref			
**CRP**							
High (≥5.485)	16 (94.1%)	9 (64.3%)	25 (80.6%)	8.89 (0.89-88.40)	0.067[b]	**Not analysed**	
Low (<5.485)	1 (5.9%)	5 (35.7%)	6 (19.4%)	Ref			
**Procalcitonin**							
High (≥2.72)	6 (35.3%)	0	6 (19.4%)	n/a	**0.021**[b]	**Not analysed**	
Low (<2.72)	11 (64.7%)	14 (100%)	24 (80.6%)	Ref			
**CRP/Alb Ratio**							
High (≥1.295)	16 (94.1%)	9 (64.3%)	25 (80.6%)	8.89 (0.89-88.40)	0.067[b]	**Not analysed**	
Low (<1.295)	1 (5.9%)	5 (35.7%)	6 (19.4%)	Ref			
**CRP/PCT Ratio**							

High (≥880.665)	2 (15.4%)	0	2 (8.3%)	n/a	0.482[b]	**Not analysed**	
Low (<880.665)	11 (84.6%)	11 (100%)	22 (91.7%)	Ref			

[a]$\chi 2$ test; [b]Fisher's exact test; OR was obtained from the Mantel-Haenszel common odds ratio estimate; [c] variables with p-value ≤0.25 was eligible to enter multivariate analysis after bivariate analysis, except variables with n/a results for their OR. Only variables with the same sample size (n=86) were included in this analysis; [d]multivariate analysis using the backward model; "n/a (not applicable)" denoted incalculably OR due to the presence of invalid (null) data in the 2 x 2 table; percent values (%) were calculated as a percentage of the column total.

Abbreviations: BAN, body mass index, albumin and neutrophil-lymphocyte ratio; BLR, basophil-to-monocyte ratio; CRP, C-reactive protein; CRP/Alb ratio, C-reactive protein-to-albumin ratio; CRP/PCT ratio, C-reactive protein-to-procalcitonin ratio; dNLR, derived neutrophil-to-lymphocyte ratio; ESR, erythrocyte sedimentation rate; HPR, haemoglobin-to-platelet ratio; LMR, lymphocyte-to-monocyte ratio; LNM, lymph node metastasis; LPR, leukocyte-to-platelet ratio; mGPS, modified Glasgow Prognostic Score; NLR, neutrophil-to-lymphocyte ratio; NMR, neutrophil-to-monocyte ratio; PCT, procalcitonin; PLR, platelet-to-lymphocyte ratio; PNI, prognostic nutritional index; Ref, reference; SII, systemic immune-inflammation index.
