# Supplementary material for: The Utility of Pre-Treatment Inflammation Markers as Associative Factors to the Adverse Outcomes of Vulvar Cancer: A Study on Staging, Nodal Involvement, and Metastasis Models
Source: J Clin Med. 2022 Dec 22;12(1):96. doi: 10.3390/jcm12010096 (PMC9821387; doi:10.3390/jcm12010096)
Supplement: Supplementary file 1 [file jcm-12-00096-s001.zip › 5. Table S5. Details bivariate and multivariate analysis of DM models.pdf]

Table S5: Detailed performance of inflammatory markers using their tailored cut-offs associated with distant metastasis in bivariate and multivariate analysis

Inflammatory markers	Distant metastasis		Total	Bivariate analysis		Multivariate analysis	
	DM (+)	DM (-)		Unadjusted OR (95%CI)	p-value	Adjusted OR (95%CI)	p-value
**LPR**							
High (≥34.15)	18 (56.3%)	20 (37.0%)	38 (44.2%)	2.19 (0.90-5.32)	0.083[a,c]	1.06 (0.30-3.81)	0.922[d]
Low (<34.15)	14 (43.8%)	34 (63.0%	48 (55.8%)	Ref			
**NLR**							
High (≥5.67)	22 (68.8%)	25 (46.3%)	47 (54.7%)	2.55 (1.02-6.40)	**0.043[a,c]**	0.71 (0.17-3.03)	0.648[d]
Low (<5.67)	10 (31.3%)	29 (53.7%)	39 (45.3%)	Ref			
**dNLR**							
High (≥3.455)	21 (65.6%)	25 (46.3%)	46 (53.5%)	2.21 (0.90-5.47)	0.082[a,c]	0.84 (0.12-5.86)	0.859[d]
Low (<3.455)	11 (34.4%)	29 (53.7%)	40 (46.5%)	Ref			
**NMR**							
High (≥9.535)	21 (65.6%)	33 (61.1%)	54 (62.8%)	1.21 (0.49-3.02)	0.676[a]	**Not analysed**	
Low (<9.535)	11 (34.4%)	21 (38.9%)	32 (37.2%)	Ref			
**PLR**							
High (≥223.965)	22 (68.8%)	28 (51.9%)	50 (58.1%)	2.04 (0.81-5.12)	0.125[a,c]	0.81 (0.22-2.93)	0.747[d]
Low (<223.965)	10 (31.2%)	26 (48.1%)	36 (41.9%)	Ref			
**LMR**							
Low (≤2.34)	26 (81.3%)	28 (51.9%)	54 (62.8%)	4.02 (1.42-11.34)	**0.006[a,c]**	1.94 (0.51-7.45)	0.332[d]
High (>2.34)	6 (18.8%)	26 (48.1%)	32 (37.2%)	Ref			
**BLR**							
High (≥0.035)	21 (65.6%)	16 (29.6%)	37 (43.0%)	4.53 (1.78-11.54)	**0.001[a,c]**	5.67 (2.02-15.87)	**0.001[d]**
Low (<0.035)	11 (34.4%)	38 (70.4%)	49 (57.0%)	Ref			
**SII**							
High (≥1348.115)	27 (84.4%)	33 (61.1%)	60 (69.8%)	3.44 (1.14-10.32)	**0.023[a,c]**	0.89 (0.07-11.12)	0.930[d]
Low (<1348.115)	5 (15.6%)	21 (38.9%)	26 (30.2%)	Ref			
**BAN Score**							
Low (≤183.84)	27 (84.4%)	32 (59.3%)	59 (68.6%)	3.73 (1.24-11.13)	**0.015[a,c]**	1.34 (0.18-9.90)	0.772[d]
High (>183.84)	5 (15.6%)	22 (40.7%)	27 (31.4%)	Ref			
**HPR**							
Low (≤0.235)	12 (37.5%)	12 (22.2%)	24 (27.9%)	2.10 (0.80-5.49)	0.127[a,c]	1.90 (0.62-5.83)	0.260[d]
High (>0.235)	20 (62.5%)	42 (77.8%)	62 (72.1%)	Ref			
**ESR**							
High (≥84)	27 (84.4%)	29 (53.7%)	56 (65.1%)	4.65 (1.56-13.90)	**0.004[a,c]**	6.01 (1.81-19.91)	**0.003[d]**
Low (<84)	5 (15.6%)	25 (46.3%)	30 (34.9%)	Ref			
**PNI Score**							
Low (≤43.50)	24 (75.0%)	28 (51.9%)	52 (60.5%)	2.79 (1.06-7.29)	**0.034[a,c]**	0.82 (0.16-4.11)	0.810[d]
High (>43.50)	8 (25.0%)	26 (48.1%)	34 (39.5%)	Ref			
**mGPS**							
High (1-2)	11 (64.7%)	9 (64.3%)	20 (64.5%)	2.14 (0.47-9.70)	0.320[a]	**Not analysed**	
Low (0)	4 (26.7%)	7 (43.8%)	11 (35.5%)	Ref			
**CRP**							
High (≥164.4)	7 (46.7%)	1 (6.3%)	8 (25.8%)	13.12 (1.36-126.30)	**0.015[b]**	**Not analysed**	
Low (<164.4)	8 (53.3%)	15 (93.8%)	23 (74.2%)	Ref			
**Procalcitonin**							
High (≥0.16)	14 (93.3%)	9 (56.3%)	23 (74.2%)	10.89 (1.14-103.98)	**0.037[b]**	**Not analysed**	
Low (<0.16)	1 (6.7%)	7 (43.8%)	8 (25.8%)	Ref			
**CRP/Alb Ratio**							
High (≥53.245)	7 (46.7%)	2 (12.5%)	9 (29.0%)	6.12 (1.01-36.89)	**0.036[a]**	**Not analysed**	
Low (<53.245)	8 (53.3%)	14 (87.5%)	22 (71.0%)	Ref			
**CRP/PCT Ratio**							

High (≥122.525)	8 (66.7%)	5 (41.7%)	13 (54.2%)	2.80 (0.53-14.73)	0.219[a]	**Not analysed**	
Low (<122.525)	4 (33.3%)	7 (58.3%)	11 (45.8%)	Ref			

[a]$\chi 2$ test; [b]Fisher's exact test; OR was obtained from the Mantel-Haenszel common odds ratio estimate; [c]variables with p-value ≤0.25 was eligible to enter multivariate analysis after bivariate analysis, but only those with the same sample sizes (n=86); [d]multivariate analysis using the backward model; "n/a (not applicable)" denoted incalculably OR due to the presence of invalid (null) data in the 2 x 2 table; percent values (%) were calculated as a percentage of the column total.

Abbreviations: BAN, body mass index, albumin and neutrophil-lymphocyte ratio; BLR, basophil-to-monocyte ratio; CRP, C-reactive protein; CRP/Alb ratio, C-reactive protein-to-albumin ratio; CRP/PCT ratio, C-reactive protein-to-procalcitonin ratio; dNLR, derived neutrophil-to-lymphocyte ratio; ESR, erythrocyte sedimentation rate; HPR, haemoglobin-to-platelet ratio; LMR, lymphocyte-to-monocyte ratio; LPR, leukocyte-to-platelet ratio; mGPS, modified Glasgow Prognostic Score; NLR, neutrophil-to-lymphocyte ratio; NMR, neutrophil-to-monocyte ratio; PCT, procalcitonin; PLR, platelet-to-lymphocyte ratio; PNI, prognostic nutritional index; Ref, reference; SII, systemic immune-inflammation index.
