# Supplementary material for: The Utility of Pre-Treatment Inflammation Markers as Associative Factors to the Adverse Outcomes of Vulvar Cancer: A Study on Staging, Nodal Involvement, and Metastasis Models
Source: J Clin Med. 2022 Dec 22;12(1):96. doi: 10.3390/jcm12010096 (PMC9821387; doi:10.3390/jcm12010096)
Supplement: Supplementary file 1 [file jcm-12-00096-s001.zip › 6. Figure S1. ROC analysis Cutoffs for Clinical Staging Model.pdf]

Figure S1: The determination of inflammatory surrogate markers cut off using the area under an ROC curve (AUC) associated with clinical staging

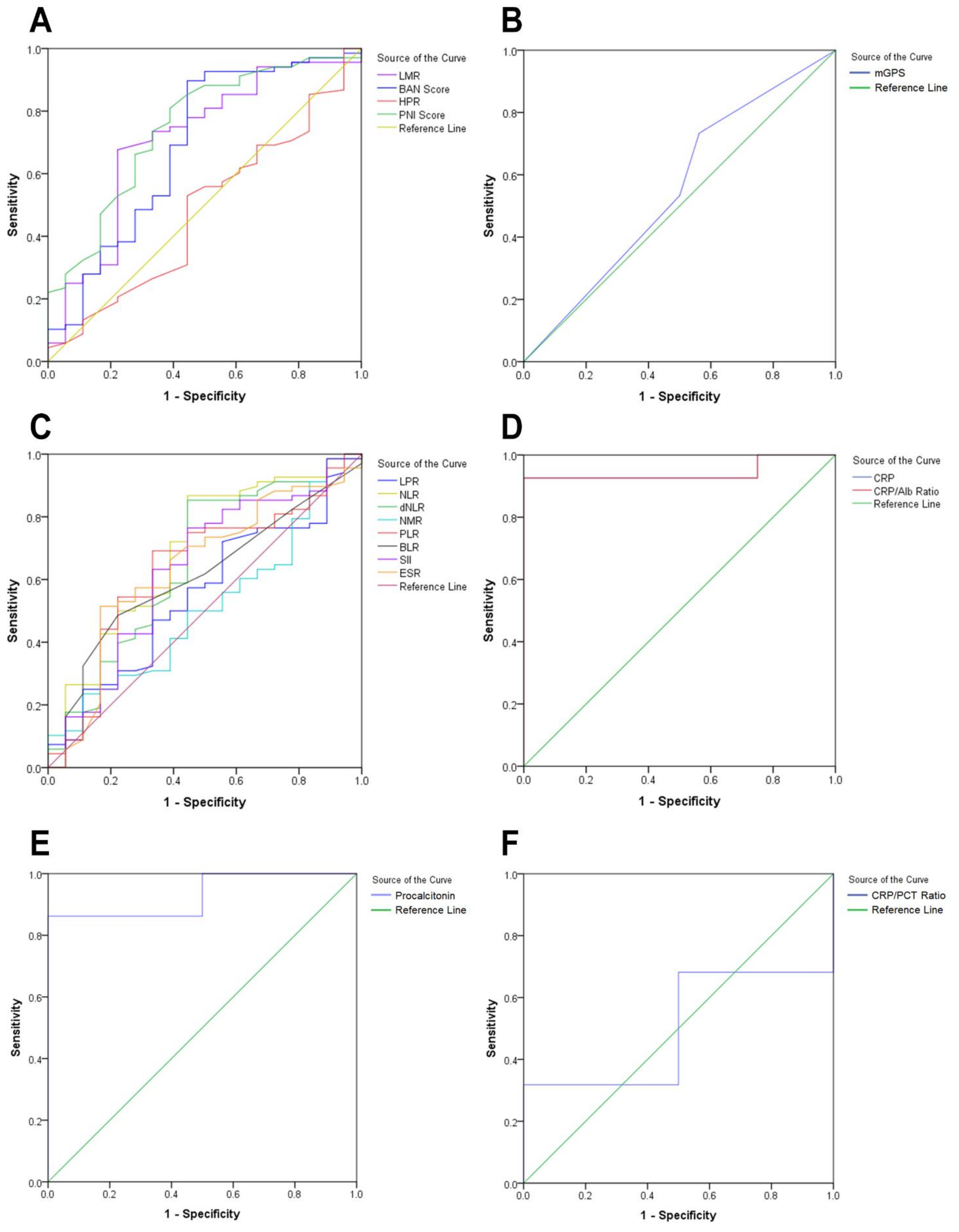

The determination of inflammatory surrogate markers' cut-off using the AUC associated with clinical staging. Graph A displays the ROC analysis of markers representing their point of interest by smaller results than the cut-off (a value below the cut-off indicates advanced disease). Graphs B

to F belongs to the ROC analysis of markers that figure their favoured cases by results below the cut-off (the case with a value above the cut-off represents advanced-stage cancer). Analysis was done separately concerning ROC's testing direction and different marker sample sizes.

Abbreviations: ROC, receiver operating characteristic; AUC, area under the ROC curve; mGPS, modified Glasgow Prognostic Score; LPR, leukocyte-to-platelet ratio; NLR, neutrophil-to-lymphocyte ratio; dNLR, derived neutrophil-to-lymphocyte ratio; NMR, neutrophil-to-monocyte ratio; PLR, platelet-to-monocyte ratio; LMR, lymphocyte-to-monocyte ratio; BLR, basophil-to-monocyte ratio; SII, systemic immune-inflammation index; BAN score, Scoring based on body-mass-index, albumin and neutrophil-lymphocyte ratio; HPR, haemoglobin-to-platelet ratio; ESR, erythrocyte sedimentation rate; CRP, C-reactive protein; CRP/Alb ratio, C-reactive protein-to-albumin ratio; CRP/PCT ratio, C-reactive protein-to-procalcitonin ratio; PNI, prognostic nutritional index.
